# T细胞受体基因重排在辅助诊断T细胞大颗粒淋巴细胞白血病中的价值

**DOI:** 10.3760/cma.j.cn121090-20250808-00366

**Published:** 2026-01

**Authors:** 莉颖 朱, 慧敏 金, 云娟 吴, 祯 郭, 琰 王, 海荣 仇, 雨洁 吴, 蕾 曹, 磊 范, 建勇 李, 纯 乔

**Affiliations:** 1 南京医科大学第一附属医院、江苏省人民医院血液科淋巴瘤中心，南京 210029 Lymphoma Center, Department of Hematology, the First Affiliated Hospital of Nanjing Medical University, Jiangsu Province Hospital, Nanjing 210029, China; 2 南京医科大学第一附属医院、江苏省人民医院风湿免疫科，南京 210029 Department of Rheumatology, the First Affiliated Hospital of Nanjing Medical University, Jiangsu Province Hospital, Nanjing 210029, China

**Keywords:** 白血病，大颗粒淋巴细胞, T细胞受体基因重排, TCRβ链可变区亚家族, STAT3基因突变, Leukemia, large granular lymphocytic, T-cell receptor gene rearrangement, TCRVβ subfamily, STAT3 gene mutation

## Abstract

**目的:**

探讨T细胞受体（TCR）基因重排在以T细胞大颗粒淋巴细胞白血病（T-LGLL）为主的T细胞异常克隆性疾病中的特点和辅助诊断价值。

**方法:**

纳入江苏省人民医院2011年9月至2023年11月初诊的T-LGLL患者103例，以再生障碍性贫血（AA）患者（18例）和纯红细胞再生障碍（PRCA）患者（3例）为再障组的患者21例，系统性红斑狼疮（SLE）患者111例以及健康对照30名。采用聚合酶链反应（PCR）-毛细管电泳法检测TCR基因重排，流式细胞术（FCM）检测TCRβ链可变区（TCRVβ）亚家族和TCRβ链恒定区结构域蛋白1（TRBC1），对比三种检测方法对上述疾病的辅助诊断价值。同时采用Sanger测序对T-LGLL患者进行STAT3基因突变检测，分析患者的突变特点及临床特征。

**结果:**

在T-LGLL患者中，男57例、女46例，中位年龄为61（28～81）岁。其中97.1％（100/103）的患者TCR基因重排阳性，检出率最高的为TCRβ基因重排（95.1％，98/103），分为TCRβ（V-J）（42.9％，42/98）、TCRβ（D-J）（15.3％，15/98）以及同时发生上述2种TCRβ基因重排（41.8％，41/98）；其次为TCRγ基因重排（47.6％，49/103）和TCRδ基因重排（4.9％，5/103）。TCRVβ和TRBC1检出率分别为89.5％（51/57）和73.5％（25/34），TRBC1的检出率低于TCR基因重排（*P*＝0.004）。再障组、SLE组和健康对照组的TCR基因重排检出率分别为19.0％、6.3％和3.3％。与健康对照组和SLE组患者相比，T-LGLL患者的HGB、RBC及ANC均较低，而淋巴细胞绝对计数（ALC）较高（*P*值均<0.01）。与再障组相比，T-LGLL患者WBC、ALC和PLT均较高（*P*值均<0.001）。相较于再障组、SLE组和健康对照组，T-LGLL患者的年龄更大且TCR基因重排检出率较高（*P*值均<0.01）。在T-LGLL中，与TCRβ（D-J）基因重排阴性患者相比，阳性患者合并自身免疫性疾病（3.6％对19.1％，*P*＝0.011）以及HGB≥100 g/L（33.9％对63.8％，*P*＝0.002）的比例更低，而B症状的发生率更高（64.3％对38.3％，*P*＝0.009）；TCRγ基因重排阳性患者的STAT3基因突变发生率增高（58.3％对30.2％，*P*＝0.012）。T-LGLL患者的中位随访时间为43（3～178）个月，总生存率为95.1％（98/103），各TCR克隆性基因重排患者的总生存期差异无统计学意义（*P*值均>0.05）。

**结论:**

TCR基因重排对T细胞克隆的检出率高于TCRVβ和TRBC1，TCRβ（D-J）基因重排阳性的患者B症状的发生率高、而合并自身免疫性疾病和HGB≥100 g/L比例低，且TCRγ基因重排阳性患者更易发生STAT3基因突变，这些特点皆有助于鉴别诊断T-LGLL。

## Abstract (en)

**Objective:**

To explore the characteristics and auxiliary diagnostic value of T-cell receptor（TCR）gene rearrangement in T-cell abnormal clonal diseases, including T-cell large granular lymphocytic leukemia（T-LGLL）.

**Methods:**

Altogether, 103 newly diagnosed patients with T-LGLL, 18 with aplastic anemia（AA）, 3 with pure red cell aplasia, 111 with systemic lupus erythematosus（SLE）, and 30 healthy controls admitted at Jiangsu Province Hospital from September 2011 to November 2023 were enrolled. TCR gene rearrangement was detected by polymerase chain reaction and capillary electrophoresis, and TCRβ chain variable region（TCRVβ）subfamily and TCRβ chain constant region 1（TRBC1）were detected by flow cytometry. The auxiliary diagnostic value of these three detection methods for T-LGLL was compared. Meanwhile, Sanger sequencing was used to detect STAT3 gene mutations in T-LGLL patients, and the mutation characteristics and clinical features of these patients were analyzed.

**Results:**

In T-LGLL patients, 57 were men and 46 were women, and the patients' median age was 61（28–81）years. Altogether, 97.1％（100/103）of the patients were positive for TCR gene rearrangement, and TCRβ gene rearrangement showed the highest incidence（95.1％, 98/103）. TCRβ gene rearrangements were further classified into TCRβ（V-J）（42.9％, 42/98）, TCRβ（D-J）（15.3％, 15/98）, and copositive for two TCRβ gene rearrangements（41.8％, 41/98）. This was followed by TCRγ（47.6％, 49/103）and TCRδ（4.9％, 5/103）gene rearrangements. The detection rates of TCRVβ and TRBC1 were 89.5％（51/57）and 73.5％（25/34）, respectively. The detection rate of TRBC1 was significantly lower than that of TCR gene rearrangement（*P*＝0.004）. The detection rates of TCR gene rearrangement in the AA, SLE, and healthy control groups were 19.0％, 6.3％, and 3.3％, respectively. Compared with healthy controls and SLE patients, the T-LGLL patients had significantly lower HGB, RBC, and ANC but significantly higher absolute lymphocyte count（ALC）（all *P*<0.01）. Compared with the AA group, the white blood cell count, ALC, and platelet counts of the T-LGLL patients were significantly higher（all *P*<0.001）. Compared with the AA, SLE, and healthy control groups, the T-LGLL patients were older and had a significantly higher detection rate of TCR gene rearrangement（all *P*<0.05）. Within the T-LGLL cohort, patients with positive TCRβ（D-J）gene rearrangement had significantly lower incidence of autoimmune diseases（3.6％ *vs* 19.1％, *P*＝0.011）and HGB of ≥100 g/L（33.9％*vs* 63.8％, *P*＝0.002）, but a significantly higher incidence of B symptoms（64.3％ *vs* 38.3％, *P*＝0.009）, than those patients without this rearrangement. Meanwhile, patients with positive TCRγ gene rearrangement had significantly higher incidence of STAT3 gene mutations（58.3％ *vs* 30.2％, *P*＝0.012）. The median follow-up time and overall survival（OS）rate of T-LGLL patients were 43（3–178）months and 95.1％（98/103）, respectively. No significant difference in the OS was observed among the patients with different types of TCR gene clonal rearrangements（all *P*>0.05）.

**Conclusion:**

The detection rate of TCR gene rearrangement for T-cell clones is higher than that of TCRVβ and TRBC1. Patients with positive TCRβ（D-J）gene rearrangement have a higher incidence of B symptoms, lower incidence of autoimmune diseases, and lower HGB levels. Patients with positive TCRγ gene rearrangement are more likely to develop STAT3 gene mutation. These characteristics may be helpful in the differential diagnosis of T-LGLL.

T细胞大颗粒淋巴细胞白血病（T-LGLL）是一种以克隆性细胞毒性T淋巴细胞（CTL）异常增殖为特征的疾病[1]，患者常表现为中性粒细胞减少、贫血、血小板减少或合并自身免疫性疾病等[2]。T-LGLL分为2种临床亚型即CD8^+^ T-LGLL（70％）和CD4^+^ T-LGLL（30％），前者较后者更常见[3]。根据T细胞受体（TCR）链的表达，T-LGLL可以划分为α/β（95％）和γ/δ（5％）亚型[4]–[5]。基于流式细胞术（FCM）的TCRβ链恒定区结构域蛋白1（TRBC1）和TCRβ链可变区（TCRVβ）亚家族分析，可实现对T细胞克隆性的快速、灵敏识别[6]。TCR基因重排对T淋巴细胞增殖性疾病（T-LPD）的检出也具有高度敏感性[7]，这3种检测方法均可用于鉴定T-LPD中的克隆性T细胞[8]–[9]，但在一些非恶性疾病如自身免疫性疾病中也可检测到TCR基因重排[10]。另外，信号转导与转录激活因子3（STAT3）是JAK/STAT信号通路的一部分，参与调控细胞增殖、分化、存活和炎症免疫等[11]，该基因突变是T-LGLL患者中最常见的突变，其中Y640F是主要的突变类型，对该疾病的诊断具有重要价值[12]。本研究通过对T-LGLL等疾病患者的TCRβ、TCRγ和TCRδ基因重排方式进行分析，探讨TCR基因重排在辅助诊断T-LGLL中的价值，并分析与患者临床特点的相关性。

## 病例与方法

一、病例

本研究为回顾性队列研究，收集江苏省人民医院血液科2011年9月至2023年11月T-LGLL患者标本103例，以再生障碍性贫血（AA）18例和纯红细胞再生障碍（PRCA）3例为再障组（21例），系统性红斑狼疮（SLE）111例，随机选取30名同期体检健康者的外周血标本作为健康对照组。所有纳入研究的T-LGLL病例均经临床和实验室检查明确诊断，符合世界卫生组织（2022）第五版造血和淋巴组织肿瘤分类标准[13]。本研究获得南京医科大学第一附属医院伦理委员会批准（批件号：2025-SR-121.A1），豁免患者知情同意。

二、聚合酶链反应（PCR）-毛细管电泳法TCR基因重排检测

抽取骨髓或外周血标本2～4 ml，采用杭州倍沃医学科技有限公司提供的血液基因组DNA提取试剂盒（柱式法）（货号：GD2311-01）提取DNA。按照苏州云泰生物医药科技有限公司生产的TCR基因重排检测试剂盒（毛细管电泳法）（货号：SL220002）说明书进行操作，将样本、相应的阳性对照品和阴性对照品分别加入反应体系中。应用Veriti热循环仪（美国Thermo Fisher Scientific有限公司）对TCRVβ、多样区（D）β、连接区（J）β、Vγ、Jγ、Vδ、Jδ片段进行PCR扩增。反应条件：95 °C预变性7 min；95 °C变性45 s，60 °C退火45 s，72 °C延伸90 s，共40个循环；最后72 °C延伸10 min。将PCR扩增产物与甲酰胺、HIDI和Genescan-500LIZ（美国Thermo Fisher Scientific有限公司）混合，经95 °C变性5 min，4 °C反应5 min后，使用3500Dx基因分析仪（美国Thermo Fisher Scientific有限公司）进行基因片段扫描分析。TCR基因重排检测采用BIOMED-2方法，结果判断标准见文献[14]。

三、FCM检测TCRVβ亚家族和TRBC1

从骨髓或外周血标本中取（1～2）×10^5^个细胞于流式测定管中，应用多参数FCM分析细胞表面不同抗原的表达情况。TCRVβ亚家族检测按照TCRVβ试剂盒（美国Beckman Coulter有限公司，货号：IM3497）说明书操作；TRBC1单克隆荧光抗体组合方案：CD3-PerCP-Cy5.5/CD4-PE/CD8-APC-Cy7/CD2-PE-Cy7/CD5-BV421/CD7-APC/CD45-BV605/TCRγδ-APC-A750/TRBC1-FITC，以上抗体购自美国Beckman Coulter有限公司、碧迪医疗器械（上海）有限公司以及河南凯普瑞生物技术有限公司。采用Navios流式细胞仪（美国Beckman Coulter有限公司）收集1×10^5^个细胞，先以CD45/SSC圈出淋巴细胞，再用CD3^+^设门，分别分析CD4^+^和CD8^+^细胞中24种TCRVβ亚家族（Vβ1、Vβ2、Vβ3、Vβ4、Vβ5.1、Vβ5.2、Vβ5.3、Vβ7.1、Vβ7.3、Vβ8、Vβ9、Vβ11、Vβ12、Vβ13.1、Vβ13.2、Vβ13.6、Vβ14、Vβ16、Vβ17、Vβ18、Vβ20、Vβ21.3、Vβ22和Vβ23）以及TRBC1的表达情况，使用Kaluza Version 2.1（美国Beckman Coulter有限公司）分析目标细胞群体的免疫表型特点。TCRVβ亚家族判定规则：目标细胞某种TCRVβ亚家族呈限制性表达（某种亚家族阳性率>50％或超过正常参考范围上限10倍）或阴性表达（24种亚家族阳性率之和<30％）。TRBC1单相表达的判定阈值：TRBC1单相占比<15％或>85％。

四、STAT3基因突变检测

在Genebank上查找STAT3基因序列（NC-000017.11），应用Primer Express软件设计相应引物。STAT3外显子20（STAT3 exon20）正义链：5′-CAAGGTGTCCTCTACAAAGATAAAG-3′，反义链：5′-CCACTGTGTTAGACATAAAGAAGAC-3′；STAT3外显子21（STAT3 exon21）正义链：5′-AAAAGACAAAATTCTTGGCACCTCC-3′，反义链：5′-GAAATAATCTGGCATATCCCTGTGG-3′。PCR总体系20 µl，含2×Taq PCR Master Mix 10 µl、正反义引物各1 µl、cDNA模板2 µl和双蒸水6 µl。应用Veriti热循环仪（美国Thermo Fisher Scientific有限公司）对STAT3 exon20和exon21进行PCR扩增。反应条件：94 °C预变性4 min；94 °C变性30 s，58 °C退火45 s，72 °C延伸1 min，共35个循环；最后72 °C延伸10 min。PCR产物使用Sanger测序，结果与STAT3基因序列进行比对。

五、随访

随访方式为电话随访，截止时间为2024年10月31日。观察终点为总生存（OS）期，即从疾病确诊至死亡或随访结束时间。

六、统计学处理

采用SPSS 26.0和Graphpad prism 9.0统计分析软件进行数据分析，根据数据分布类型，偏态分布连续变量资料用*M*（范围）描述，组间比较采用非参数检验（Mann-Whitney *U*检验）。分类变量资料采用率或构成比表示，组间比较采用*χ*^2^检验。应用Logistic回归分析筛选影响因素，计算比值比（*OR*）和95％置信区间（*CI*）。采用Kaplan-Meier法绘制生存曲线。*P*<0.05为差异有统计学意义。

## 结果

一、基本临床特征

如表1所示，T-LGLL组（103例）中，男57例（55.3％）、女46例（44.7％），中位年龄为61（28～81）岁；再障组（21例）中，男13例（61.9％）、女8例（38.1％），中位年龄为48（15～75）岁；111例SLE患者中，男7例（6.3％）、女104例（93.7％），中位年龄为35（13～75）岁。健康对照组（30名）中，男7名（23.3％）、女23名（76.7％），中位年龄为40（25～75）岁，既往无血液病史。与健康对照组和SLE组相比，T-LGLL组患者的HGB、RBC及ANC均较低，而淋巴细胞绝对计数（ALC）较高（*P*值均<0.01）。与再障组相比，T-LGLL组患者WBC、ALC和PLT均较高（*P*值均<0.001）。相较于再障组、SLE组和健康对照组，T-LGLL组患者的年龄更大且TCR基因重排检出率较高（*P*值均<0.01）（表1）。为避免各组间年龄差异干扰TCR基因重排检出率，将疾病诊断作为自变量、TCR基因重排检出率作为因变量、年龄作为协变量，构建了Logistic回归模型。结果显示，以年龄作为协变量的*P*值均>0.05，证实了相较于再障组、SLE组和健康对照组，T-LGLL组患者TCR基因重排检出率高是独立于年龄的。

**表1:** 不同组别患者及健康对照组的基本临床特征比较

临床特征	T-LGLL组 （103例）	再障组 （21例）	SLE组 （111例）	健康对照组 （30名）	*U*/*χ*^2^值^a^	*P*值^a^	*U*/*χ*^2^值^b^	*P*值^b^	*U*/*χ*^2^值^c^	*P*值^c^
性别（男/女）	57/46	13/8	7/104	7/23	0.306	0.580	61.276	<0.001	9.534	0.002
年龄［岁，*M*（范围）］	61（28～81）	48（15～75）	35（13～75）	40（25～75）	636.5	0.003	2 461.5	<0.001	731.5	<0.001
HGB［g/L，*M*（范围）］	93（26～160）	71（43～164）	116（51～153）	131（108～156）	882.0	0.184	3 398.5	<0.001	469.5	<0.001
RBC［×10^12^/L，*M*（范围）］	2.7（0.7～5.6）	2.2（1.1～5.4）	4.1（1.3～5.3）	4.4（3.8～5.7）	853.0	0.128	2 863.5	<0.001	461.0	<0.001
WBC［×10^9^/L，*M*（范围）］	5.6（0.5～35.8）	2.6（0.5～6.4）	5.8（1.1～22.6）	6.3（3.7～8.7）	446.5	<0.001	5 537.0	0.692	1 317.0	0.220
ALC［×10^9^/L，*M*（范围）］	2.6（0.2～23.3）	1.1（0.3～2.7）	1.2（0.1～3.7）	2.0（1.2～3.5）	449.0	<0.001	2 365.5	<0.001	1 048.0	0.007
ANC［×10^9^/L，*M*（范围）］	1.4（0.2～6.7）	1.2（0～4.4）	3.8（0.6～19.5）	3.8（1.8～5.9）	894.0	0.212	1 746.5	<0.001	397.5	<0.001
PLT［×10^9^/L，*M*（范围）］	205（13～465）	15（2～276）	195（1～617）	209（103～354）	362.0	<0.001	5 563.5	0.735	1 363.5	0.328
TCR基因重排［例（％）］	100（97.1）	4（19.0）	7（6.3）	1（3.3）	78.532	<0.001	176.115	<0.001	111.768	<0.001

**注** T-LGLL：T细胞大颗粒淋巴细胞白血病；再障：包括再生障碍性贫血和纯红细胞再生障碍；SLE：系统性红斑狼疮；HGB：血红蛋白；RBC：红细胞计数；WBC：白细胞计数；ALC：淋巴细胞绝对计数；ANC：中性粒细胞绝对计数；PLT：血小板计数；TCR：T细胞受体；^a^T-LGLL组与再障组比较；^b^T-LGLL组与SLE组比较；^c^T-LGLL组与健康对照组比较

二、T-LGLL患者的免疫表型以及不同组别的TCR基因重排结果

T-LGLL患者细胞的免疫表型包括CD3^+^ CD4^-^ CD8^+^（86.4％，89/103）、CD3^+^CD4^+^CD8^-^（3.9％，4/103）、CD3^+^ CD4^+^ CD8^+^（2.9％，3/103）和CD3^+^ CD4^-^ CD8^-^（6.8％，7/103），CD57阳性表达患者占66.7％（52/78）。对38例患者进行TCRαβ/TCRγδ表型鉴定，其中TCRαβ表型占92.1％（35/38），TCRγδ表型占7.9％（3/38）。

在T-LGLL患者中，97.1％（100/103）的患者TCR基因重排检测阳性，其中检出率最高的为TCRβ基因重排（95.1％，98/103），其次为TCRγ基因重排（47.6％，49/103）和TCRδ基因重排（4.9％，5/103）。TCRβ基因重排方式分为TCRβ（V-J）（80.6％，83/103）和TCRβ（D-J）（54.4％，56/103），包括TCRβ（V-J）单克隆基因重排（42.9％，42/98）、TCRβ（D-J）单克隆基因重排（15.3％，15/98）以及TCRβ（V-J）和TCRβ（D-J）基因重排同时阳性（41.8％，41/98）。在TCRαβ表型和TCRγδ表型的T-LGLL患者中，TCRβ基因重排阳性率分别为94.3％（33/35）和100％（3/3）。19.0％（4/21）的再障组、6.3％（7/111）的SLE组以及3.3％（1/30）的健康对照组具有TCR基因重排的单克隆模式。在再障组中，TCRβ和TCRγ基因重排阳性率均为9.5％（2/21），未检测到TCRδ基因重排。在SLE组患者中TCRγ基因重排阳性率（4.5％，5/111）高于TCRβ（V-J）基因重排（1.8％，2/111）和TCRδ基因重排（0.9％，1/111），未检测到TCRβ（D-J）基因重排。健康对照组中仅有1名检出TCR基因重排阳性，重排类型为TCRγ。

三、TCR基因重排与临床生物学特征

在103例T-LGLL患者中，11例（10.7％）患者合并自身免疫性疾病，包括类风湿性关节炎（RA）7例、干燥综合征（SS）3例、自身免疫性溶血性贫血（AIHA）3例，其中2例同时合并RA和SS。与TCRβ（D-J）基因重排阴性患者相比，阳性患者合并自身免疫性疾病（3.6％对19.1％，*P*＝0.011）以及HGB≥100 g/L的比例（33.9％对63.8％，*P*＝0.002）更低，B症状的发生率更高（64.3％对38.3％，*P*＝0.009）。TCR基因重排与性别、是否合并PRCA、ANC、PLT等其他临床特征无相关性（*P*值均>0.05，表2）。

**表2:** 不同TCR基因重排类型的T-LGLL患者临床特征比较［例（％）］

临床特征	TCRβ（V-J）			TCRβ（D-J）			TCRγ			TCRδ		
	阳性（83例）	阴性（20例）	*χ*^2^/*P*值	阳性（56例）	阴性（47例）	*χ*^2^/*P*值	阳性（49例）	阴性（54例）	*χ*^2^/*P*值	阳性（5例）	阴性（98例）	*χ*^2^/*P*值
性别			1.074/0.300			0.627/0.428			1.530/0.216			0.061/0.806
男（57例）	48（57.8）	9（45.0）		29（51.8）	28（59.6）		24（49.0）	33（61.1）		2（40.0）	55（56.1）	
女（46例）	35（42.2）	11（55.0）		27（48.2）	19（40.4）		25（51.0）	21（38.9）		3（60.0）	43（43.9）	
是否合并自身免疫性疾病			0/>0.05			6.500/0.011			0.620/0.431			0/>0.05
是（11例）	9（10.8）	2（10.0）		2（3.6）	9（19.1）		4（8.2）	7（13.0）		1（20.0）	10（10.2）	
否（92例）	74（89.2）	18（90.0）		54（96.4）	38（80.9）		45（91.8）	47（87.0）		4（80.0）	88（89.8）	
是否合并PRCA			0.427/0.514			1.051/0.305			0.110/0.741			0/>0.05
是（32例）	27（32.5）	5（25.0）		15（26.8）	17（36.2）		16（32.7）	16（29.6）		2（40.0）	30（30.6）	
否（71例）	56（67.5）	15（75.0）		41（73.2）	30（63.8）		33（67.3）	38（70.4）		3（60.0）	68（69.4）	
ANC			0/>0.05			0.883/0.347			0.032/0.858			0/>0.05
ANC<0.5×10^9^/L（12例）	10（12.0）	2（10.0）		5（8.9）	7（14.9）		6（12.2）	6（11.1）		1（20.0）	11（11.2）	
ANC≥0.5×10^9^/L（91例）	73（88.0）	18（90.0）		51（91.1）	40（85.1）		43（87.8）	48（88.9）		4（80.0）	87（88.8）	
HGB			1.573/0.210			9.160/0.002			2.900/0.089			0.651/0.420
HGB<100 g/L（54例）	41（49.4）	13（65.0）		37（66.1）	17（36.2）		30（61.2）	24（44.4）		4（80.0）	50（51.0）	
HGB≥100 g/L（49例）	42（50.6）	7（35.0）		19（33.9）	30（63.8）		19（38.8）	30（55.6）		1（20.0）	48（49.0）	
PLT			0/>0.05			0.067/0.796			0.651/0.420			0/>0.05
PLT<50×10^9^/L（5例）	4（4.8）	1（5.0）		3（5.4）	2（4.3）		1（2.0）	4（7.4）		0（0）	5（5.1）	
PLT≥50×10^9^/L（98例）	79（95.2）	19（95.0）		53（94.6）	45（95.7）		48（98.0）	50（92.6）		5（100）	93（94.9）	
是否伴有B症状			3.022/0.082			6.919/0.009			0.833/0.361			3.793/0.051
是（54例）	47（56.6）	7（35.0）		36（64.3）	18（38.3）		28（57.1）	26（48.1）		0（0）	54（55.1）	
否（49例）	36（43.4）	13（65.0）		20（35.7）	29（61.7）		21（42.9）	28（51.9）		5（100）	44（44.9）	
STAT3基因突变^a^			2.024/0.155			2.135/0.144			6.312/0.012			0.062/0.804
阳性（34例）	30（46.9）	4（26.7）		20（51.3）	14（35.0）		21（58.3）	13（30.2）		2（66.7）	32（42.1）	
阴性（45例）	34（53.1）	11（73.3）		19（48.7）	26（65.0）		15（41.7）	30（69.8）		1（33.3）	44（57.9）	

**注** TCR：T细胞受体；T-LGLL：T细胞大颗粒淋巴细胞白血病；V：可变区；J：连接区；D：多样区；PRCA：纯红细胞再生障碍；ANC：中性粒细胞绝对计数；HGB：血红蛋白；PLT：血小板计数；^a^仅79例T-LGLL患者行Sanger测序检测STAT3基因突变情况

采用Sanger测序对79例T-LGLL患者进行STAT3基因突变检测，43.0％（34/79）的患者为STAT3基因突变阳性，其中Y640F 19例（19/34，55.9％）。与阴性患者相比，TCRγ基因重排阳性患者的STAT3基因突变发生率更高（58.3％对30.2％，*P*＝0.012）。其他类型的TCR基因重排均与STAT3基因突变无明显相关性（*P*值均>0.05）。

四、TCRVβ亚家族和TRBC1的表达特点

应用FCM对57例T-LGLL患者进行TCRVβ检测，结果发现24种TCRVβ亚家族中呈限制性表达或阴性表达者51例（89.5％），其中克隆性T淋巴细胞发生率最高的亚家族成员是TCRVβ2（14.0％，8/57），其次为TCRVβ13.2（8.8％，5/57）和TCRVβ14（8.8％，5/57）。在34例T-LGLL患者中检测TRBC1表达情况，其中25例T细胞呈TRBC1单相表达模式（高表达或低表达），检出率为73.5％。相较于TCRVβ和TRBC1检测方法，PCR-毛细管电泳法TCR基因重排的阳性率更高，分别为98.2％（56/57）（*P*>0.05）和100％（34/34）（*P*＝0.004）。

五、T-LGLL患者临床生物学特征的影响因素分析

在T-LGLL患者中，将TCR基因重排类型作为自变量，分别以差异有统计学意义的临床特征为因变量，年龄为协变量进行Logistic回归分析，结果显示，TCRβ（D-J）基因重排是T-LGLL患者较少合并自身免疫性疾病（*OR*＝0.127，95％ *CI*：0.022～0.743，*P*＝0.022）、HGB减低（*OR*＝2.852，95％ *CI*：1.212～6.713，*P*＝0.016）和B症状发生（*OR*＝4.611，95％ *CI*：1.794～11.846，*P*＝0.002）的影响因素，TCRγ基因重排是T-LGLL患者STAT3基因突变发生率增高（*OR*＝4.763，95％ *CI*：1.313～17.287，*P*＝0.018）的影响因素。年龄作为协变量，均未见差异有统计学意义（*P*值均>0.05）。

六、TCR基因重排与预后

103例T-LGLL患者中位随访时间为43（3～178）个月，OS率为95.1％（98/103）。各TCR基因克隆性重排患者的中位OS期均未达到且差异无统计学意义（*P*值均>0.05）。5例死亡患者均TCRβ基因重排阳性并伴有B症状，其中4例TCRβ（V-J）和TCRβ（D-J）基因重排同时阳性，3例患者可检出TCRγ基因重排。

## 讨论

T-LGLL是一类慢性淋巴细胞增殖性疾病，患者的中位生存期约为10年，常见的临床表现包括贫血、中性粒细胞减少、持续性大颗粒淋巴细胞增多等[15]。T-LGLL的发病机制尚未完全明确，一般认为是由抗原长期刺激免疫系统导致CTL的单克隆增殖所致，因此T细胞的单克隆表达是诊断T-LGLL的重要依据。目前TCR基因重排是评估T细胞克隆性的重要分子学方法[16]，广泛应用于T-LPD、急性T淋巴细胞白血病、自身免疫性疾病和感染性疾病的诊断[17]。Hsieh等[18]研究发现94％的T-LGLL患者出现TCR克隆性基因重排。此外，有研究发现TCRγ基因重排在AIHA中发生率较高[19]。本研究回顾性分析了以T-LGLL为主的T细胞异常增殖性疾病患者的临床特征和生存情况，分析了TCR基因重排在诊断T-LGLL中的价值以及不同重排方式与患者临床特点的相关性。

T-LGLL患者常合并发生自身免疫性疾病，包括血液学（如AIHA、免疫性血小板减少症等）和非血液学（如RA、SLE、SS等）疾病[20]。SLE是自身免疫介导的慢性弥漫性结缔组织病，以肾脏、血液系统和神经系统等多部位受累为主要特征，由于免疫功能异常，感染是其预后不良的常见原因，其中细菌感染发生率较高[21]，因此患者的ANC可出现增高。Magnano等[22]发现约50％的LGLL患者淋巴细胞计数轻度升高，部分患者出现贫血和ANC减少，而PLT减少较为少见。本研究中，与SLE组和健康对照组相比，T-LGLL组患者RBC、HGB、ANC减少，而ALC增多；与再障组相比，T-LGLL组患者WBC、ALC和PLT增多，与报道结果一致。Liu等[2]和Marchand等[23]研究发现，T-LGLL易发于老年患者，中位诊断年龄分别为59岁和66.5岁。本研究结果显示，与再障、SLE和健康对照组相比，T-LGLL组老年患者比例更高，TCR基因重排检出率更高，与上述研究结论一致。为避免各组间年龄差异干扰TCR基因重排阳性率，以年龄作为协变量构建了Logistic回归模型，结果证实T-LGLL组TCR基因重排阳性率高是独立于年龄的。

TCR是由2个二硫键连接的跨膜蛋白（1条α链和1条β链，或1条γ链和1条δ链）组成的异源二聚体[24]，其克隆性可通过多重PCR进行基因重排分析，或多参数FCM进行TCRVβ亚家族和TRBC1检测。Hsieh等[18]对T-LGLL患者TCRVβ与TCR基因重排结果的比较研究发现，FCM法TCRVβ分析检测克隆性T淋巴细胞的敏感性略高于TCR基因重排。而在本研究中，PCR-毛细管电泳法TCR基因克隆性基因重排的检出率高于FCM检测的TCRVβ和TRBC1。在不同人群中的TCR基因重排结果显示，T-LGLL组患者中检出率最高的TCR基因重排为TCRβ基因重排（95.1％）；TCRαβ表型和TCRγδ表型T-LGLL患者的TCRβ基因重排阳性率分别为94.3％和100％。T-LGLL组患者常见的TCR基因重排也可在再障、SLE和健康对照组中发现。本研究结果显示，再障组中TCRβ和TCRγ基因重排阳性率均为9.5％，SLE中以TCRγ基因重排为主（4.5％，5/111），而健康对照组中仅1名TCRγ基因重排。Sidorova等[25]对克隆性T细胞在非恶性疾病和T-LGLL鉴别诊断的研究显示，TCRβ单克隆性基因重排在T-LGLL患者中具有较高的检出率（96.7％），而在反应性淋巴细胞增多症患者、RA患者和健康个体中也存在单克隆TCR基因重排，其中阳性率最高的重排方式均为TCRγ基因重排，与本研究结果一致。TCRγ基因座缺少D片段导致互补决定区（CDR）3较短（长度为20～30 bp），连接多样性较低容易引起非恶性疾病中的阳性结果，常见于有炎症或感染的个体中，这可能是TCRγ基因重排在再障、SLE和健康对照组中阳性率较高的原因。Wang等[26]证实，在衰老和炎症的情况下可能会产生TCR基因重排阳性结果。Semenzato等[27]对不同年龄组克隆性T细胞的研究证实，随着年龄的增长，尤其是在老年人群中，T细胞克隆性基因重排的发生率更高。因此，在老年人群中或某些非恶性疾病中也可能出现TCR基因重排，需要进一步随访观察。TCR基因重排尤其是TCRβ基因重排，对于恶性T淋巴细胞增多的鉴别诊断具有重要价值。

本研究结果显示TCRβ（D-J）基因重排阳性患者出现HGB减低和B症状的概率高，但自身免疫性疾病的发生率低。除了V、J和恒定区（C）外，TCRβ还具有独特的D片段[28]，这可能是TCRβ（D-J）基因重排与一些临床特征存在相关性的分子基础。研究发现，几乎所有的T-LGLL病例中都存在JAK-STAT通路激活，并且在约50％的患者中发现了STAT3基因突变[29]。在本研究中43.0％的T-LGLL患者具有STAT3突变，与既往研究结果一致。由于一代测序的灵敏度低于二代测序，且部分T-LGLL患者存在大颗粒淋巴细胞比例较低的情况可能影响了突变的检出率。进一步分析发现TCRγ基因重排阳性患者STAT3基因突变率显著增高，既往Teramo等[30]对T-LGLL的基因图谱研究也表明，STAT3在CD8^+^ T-LGLL和TCRγδ表型T-LGLL患者中的突变率较高。本研究以年龄作为协变量进行Logistic回归分析，再次证实TCRβ（D-J）基因重排是T-LGLL患者较少合并自身免疫性疾病、HGB减低和B症状发生的影响因素，TCRγ基因重排是T-LGLL患者STAT3基因突变发生率增高的影响因素。此外，本研究未发现不同类型的TCR基因重排与患者OS的相关性，且由于T-LGLL中大多数病例相对惰性[31]，本研究中仅有5例患者死亡。

综上，本研究对T-LGLL患者TCR基因重排特点分析发现，TCR基因重排对T细胞克隆的检出率高于TCRVβ和TRBC1，TCRβ（D-J）基因重排阳性的患者B症状的发生率增高、而自身免疫性疾病发生率以及HGB水平均减低，且TCRγ基因重排阳性患者更易发生STAT3基因突变。由于克隆性不等同于恶性，TCR基因重排对某些非恶性疾病的诊断不具有特异性。以TCR基因基因重排为主的分子生物学方法具有高灵敏度等特点，能够提高对判断良恶性克隆的准确率。在实际临床应用中，还需结合形态学、免疫表型和临床特征对疾病进行鉴别和综合诊断。
